# Elective peri‐operative management of adults taking glucagon‐like peptide‐1 receptor agonists, glucose‐dependent insulinotropic peptide agonists and sodium‐glucose cotransporter‐2 inhibitors: a multidisciplinary consensus statement: A consensus statement from the Association of Anaesthetists, Association of British Clinical Diabetologists, British Obesity and Metabolic Surgery Society, Centre for Perioperative Care, Joint British Diabetes Societies for Inpatient Care, Royal College of Anaesthetists, Society for Obesity and Bariatric Anaesthesia and UK Clinical Pharmacy Association

**DOI:** 10.1111/anae.16541

**Published:** 2025-01-09

**Authors:** Kariem El‐Boghdadly, Jugdeep Dhesi, Philippa Fabb, Nicholas Levy, Dileep N. Lobo, Andrew McKechnie, Omar Mustafa, Philip Newland‐Jones, Anil Patel, Dimitri J. Pournaras, Ken Clare, Ketan Dhatariya

**Affiliations:** ^1^ Department of Anaesthesia and Perioperative Care Guy's and St Thomas' NHS Foundation Trust London UK; ^2^ King's College London London UK; ^3^ Department of Ageing and Health Guy's and St Thomas' NHS Foundation Trust London UK; ^4^ Department of Anaesthesia Portsmouth Hospitals University NHS Trust Portsmouth UK; ^5^ Department of Anaesthesia and Perioperative Medicine West Suffolk NHS Foundation Trust Suffolk UK; ^6^ Division of Translational Medical Sciences, Nottingham Digestive Diseases Centre, School of Medicine University of Nottingham, Queen's Medical Centre Nottingham UK; ^7^ Division of Surgery, Perelman School of Medicine University of Pennsylvania Philadelphia PA USA; ^8^ Department of Anaesthesia Lewisham and Greenwich NHS Trust London UK; ^9^ Department of Diabetes King's College Hospital London UK; ^10^ Department of Diabetes and Endocrinology University of Southampton Southampton UK; ^11^ Department of Anaesthesia University College London London UK; ^12^ Bristol Weight Management and Bariatric Service North Bristol NHS Trust Bristol UK; ^13^ Patient Representative; ^14^ Department of Medicine Norfolk and Norwich University Hospitals NHS Foundation Trust Norwich UK; ^15^ University of East Anglia Medical School Norwich UK

**Keywords:** anaesthesia, aspiration, diabetes mellitus, gastric emptying, GLP‐1RA based treatment, SGLT2 inhibitors

## Abstract

**Introduction:**

Glucagon‐like peptide‐1 receptor agonists, dual glucose‐dependent insulinotropic peptide receptor agonists and sodium‐glucose cotransporter‐2 inhibitors are used increasingly in patients receiving peri‐operative care. These drugs may be associated with risks of peri‐operative pulmonary aspiration or euglycaemic ketoacidosis. We produced a consensus statement for the peri‐operative management of adults taking these drugs.

**Methods:**

This multidisciplinary consensus statement included surgeons, anaesthetists, physicians, pharmacists and people with lived experience relevant to these guidelines. Following the directed literature review, a three‐round modified Delphi process was conducted to generate and ratify recommendations.

**Results:**

Patients taking glucagon‐like peptide‐1 receptor agonists and dual glucose‐dependent insulinotropic peptide receptor agonists should: continue these drugs before surgery; have full risk assessment and stratification; and receive peri‐operative techniques that may mitigate risk of pulmonary aspiration before, during and after sedation or general anaesthesia. Patients taking sodium‐glucose cotransporter‐2 inhibitors should omit them the day before and the day of a procedure. All patients should have risks and mitigation strategies discussed with a shared decision‐making approach.

**Discussion:**

Until more evidence becomes available, this pragmatic, multidisciplinary consensus statement aims to support shared decision‐making and improve safety for patients taking glucagon‐like peptide‐1 receptor agonists, dual glucose‐dependent insulinotropic peptide receptor agonists and sodium‐glucose cotransporter‐2 inhibitors during the peri‐operative period.

## Introduction

Novel antihyperglycaemic drugs are used increasingly to treat people with and without diabetes mellitus to reduce cardiovascular and renal morbidity and mortality [1, 2, 3]. These include: incretin‐based hormone therapies, i.e. glucagon‐like peptide‐1 receptor agonists (GLP‐1RA);  dual glucose‐dependent insulinotropic peptide receptor agonists (GIP)/GLP‐1 RA; and sodium‐glucose cotransporter‐2 (SGLT2) inhibitors.

The most common indication for GLP‐1 RAs other than diabetes mellitus is obesity, and further indications are under investigation, including: osteoarthritis; obstructive sleep apnoea; metabolic dysfunction‐associated steatotic liver disease; and metabolic dysfunction‐associated steatohepatitis [4]. There is thus an increasing likelihood that people presenting for surgery or other procedures requiring anaesthesia will be taking these drugs. Due to evidence of delayed gastric emptying [5, 6, 7, 8], along with published reports of regurgitation and pulmonary aspiration [9, 10, 11, 12], conflicting suggestions have been made for peri‐operative management of patients taking GLP‐1RAs [13, 14, 15, 16]. There is a dearth of high‐quality evidence in this field and, therefore, uncertainty regarding the safest peri‐operative management strategies.

In addition to diabetes mellitus, SGLT2 inhibitors are also now licensed for the treatment of heart failure and chronic kidney disease in people without diabetes mellitus. These increasing indications for SGLT2 inhibitors have led to a greater number of people presenting for surgery also taking this class of medication [17]. Risks of diabetic ketoacidosis (DKA), in particular euglycaemic DKA, with glucose concentrations < 11.0 mmol.l^‐1^ (200 mg.dl^‐1^) have resulted in recent contradictory recommendations in the peri‐operative setting [18, 19, 20, 21].

Given the significant risks of peri‐operative complications, recommendations for peri‐operative management of patients taking both classes of medications have been published [13, 14, 15, 22, 23], with substantial variation in guidance [24]. However, these have been limited by: insufficient objective assessment of the available evidence base; the methods used for developing recommendations; standardised approaches to management; and institutional buy‐in. We aimed to produce a multidisciplinary consensus statement on behalf of the Association of Anaesthetists, Association of British Clinical Diabetologists, British Obesity and Metabolic Surgery Society, Centre for Perioperative Care, Joint British Diabetes Societies for Inpatient Care, Royal College of Anaesthetists and the Society for Obesity and Bariatric Anaesthesia for the elective peri‐operative management of adults taking GLP‐1RAs or SGLT2 inhibitors.

## Methods

This multidisciplinary consensus includes authors representing surgeons, anaesthetists, physicians, pharmacists and people with lived experience relevant to these guidelines. We performed a directed literature review for relevant evidence and used these data to support the development of recommendations in a modified Delphi process. In the first round, these recommendations were distributed amongst all authors who anonymously rated each as agree, disagree or revise, as well as providing anonymised comments onto a Microsoft Excel spreadsheet (Microsoft Inc., Redmond, WA, USA). Recommendations with ≥ 75% agreement proceeded unchanged into the second round; 50–74% were revised, and < 50% were deemed unsuitable for recommendation. A second round followed similar processes to the first, with anonymised comments and full voting results shared after each round. We then held a virtual round table for final agreement and ratification of recommendations. Recommendations were then shared with endorsing organisations for approval.

## Glucagon‐like peptide‐1 receptor and glucose‐dependent insulinotropic polypeptide receptor agonists

The incretin hormones GLP‐1 and GIP are secreted from the lining of the small bowel. Glucagon‐like peptide‐1 is secreted from the L cells throughout the intestinal tract, with higher concentrations in the distal ileum, and GIP from the K cells in the duodenum and jejunum, both in response to glucose in the gut lumen [25]. They cause a glucose‐dependent increase in insulin secretion from beta cells but have multiple other effects, including alteration of glucagon secretion from alpha cells: GLP‐1 reducing glucagon secretion, with GIP increasing it; and delaying gastric emptying, thus slowing the rise in postprandial glucose [8, 26, 27]. They also work centrally by activating hypothalamic satiety centres [28]. This is responsible for the predominant adverse effect of nausea seen commonly on drug initiation or dose escalation. However, the effect on satiety is also one of the reasons why these drugs are used for successful weight loss. In addition, both hormones increase insulin sensitivity, although through different mechanisms: GLP‐1 works mainly on the liver, while GIP works on skeletal muscle [27]. People with type 2 diabetes mellitus or obesity have very low plasma concentrations of incretin hormones compared with those without the condition.

The GLP‐1RAs liraglutide, semaglutide and dulaglutide are used increasingly for people with diabetes mellitus because of improvement in glycaemic control and reduction in risk of major adverse cardiovascular events (Table 1) [29, 30, 31, 32]. Whilst the results of cardiovascular outcome studies are awaited [2], the GLP‐1/GIP RA tirzepatide has been shown to significantly improve surrogate markers of cardiovascular disease in people with and without diabetes mellitus [33]. Furthermore, because of the significant benefit of tirzepatide on weight loss – a mean weight loss of over 20% of initial body weight over 72 weeks in people without diabetes mellitus – many of these drugs are also now licensed for managing obesity in people with or without diabetes mellitus [34, 35]. For these reasons, incretin‐based therapies are recommended for early use in people with type 2 diabetes mellitus with known risk factors for cardiovascular disease and/or obesity [36]. Furthermore, in the peri‐operative setting, data suggest that acute use (i.e. starting a few hours either side of a surgical intervention) is associated with: improved peri‐operative glycaemic control; lower postoperative insulin requirements; a lower risk of hypoglycaemia; and improved cardiovascular outcomes [37, 38, 39].

**Table 1:** Differences in the properties of GLP‐1RA and GIP. Adapted from [29].

	GLP‐1 receptor agonists							GIP agonist
	Exenatide (twice daily)	Lixisenatide (once daily)	Liraglutide (once daily)	Exenatide (once weekly)	Albiglutide (once weekly)	Dulaglutide (once weekly)	Semaglutide	Tirzepatide (once weekly)
Example trade name	Byetta	Lyxumia	Victoza	Byetta	Eperzan	Trulicity	Ozempic/Wegovy	Mounjaro
Administration route	SC	SC	SC	SC	SC	SC	SC (weekly) or oral	SC
**Pharmacokinetics**								
Total dose range (for diabetes and/or obesity)	10–20 μg	10–20 μg	Diabetes 0.6–1.8 mg Weight loss 0.6–3.0 mg	2 mg	30–50 mg	0.75–4.5 mg	Diabetes (weekly sc) 0.25–1 mg Diabetes (oral) 3–14 mg Weight loss (weekly sc) 0.25–2.4 mg weekly	Diabetes or weight loss 2.5–15 mg weekly
AUC; after single dose	247 pm.h^‐1^	NS	256 pm.h^‐1^	NS	465 μg.h^‐1^.ml^‐1^	14,000 ng.h^‐1^.ml^‐1^	2600 nmol.h^‐1^.l^‐1^	43,459–63,467 ng.h^‐1^.ml^‐1^
C_max_	50 pm.l^‐1^		9 nm.l^‐1^	55 pm.l^‐1^	1.74 μg.ml^‐1^	114 ng.ml^‐1^	10.3 nmol.l^‐1^	1250 ng.ml^‐1^
T_max_; h unless stated otherwise	2.1	1–3.5	10–14	6–7 weeks	3–5 days	48	24	24
Bioavailability; %	65–76		55	65–76	NS	65 (0.75 mg) 47 (1.5 mg)	0.8% (oral) 89% (SC)	80
Volume of distribution; l	28.3	100	13	28.3	11	19.2 (0.75 mg) 17.4 (1.5 mg)	8	10.3
Fraction bound to plasma protein; %	NS	55	> 98	NS	NS		> 99	> 99
Elimination half‐life; h unless stated otherwise	2.4	3	13	2.4 (as for exenatide)	5 days	4.5 days (0.75 mg) 4.7 days (1.5 mg)	7 days	5 days
Major elimination route	Renal/proteolysis	Renal/proteolysis	Metabolised	Renal/proteolysis	Metabolised	Metabolised	Metabolised	Metabolised
Dose reduction with renal impairment*	Mild RI, no. Moderate RI, caution with dose increase. ESRD/severe RI, not recommended.	Mild RI, no. Moderate RI, caution. ESRD/severe RI, not recommended.	Mild RI, no. Moderate RI, no. ESRD/severe RI, not recommended	Mild RI, no. Moderate RI, not recommended. ESRD/severe RI, not recommended	Mild RI, no. Moderate RI, no. ESRD/severe RI, not recommended	Mild RI, no. Moderate RI, no. ESRD/severe RI, not recommended	No dose change necessary (but not recommended in ESRD).	No dose change necessary (but not recommended in ESRD).
Active metabolite	No	No	No	No	No	No	No	No

GLP‐1, glucagon‐like peptide‐1; GIP, glucose‐dependent insulinotropic peptide; SC, subcutaneous; NS, not significant; AUC, area under the curve; C_max_, peak serum concentration; T_max_, time to peak serum concentration; RI, renal impairment; ESRD, end‐stage renal disease.

* Renal impairment categories are mild (estimated glomerular filtration rate (eGFR) 60–89 ml.min^‐1^.1.73 m^‐2^), moderate (eGFR 30–59 ml.min^‐1^.1.73 m^‐2^), or severe (eGFR ≤ 29 ml.min^‐1^.1.73 m^‐2^).

However, the peri‐operative use of these drugs is subject to scrutiny. Some studies suggest an association between peri‐operative GLP‐1 RA use and increased risk of pulmonary aspiration, with a reported OR (95%CI) of 10.23 (2.94–35.82) in the elective surgical setting [40], and an HR (95%CI) in the endoscopy setting of 1.33 (1.02–1.74). There are, however, conflicting data on the magnitude of these risks [41]. Regardless, it appears likely that gastric emptying is delayed in people taking GLP‐1 RAs as highlighted by the Pharmacovigilance Risk Assessment Committee of the European Medicines Agency [42].

In people using GLP‐1 RAs long‐term [43], there is less certainty in regard to the risk of pulmonary aspiration [44], particularly because the effect of tachyphylaxis on gastric emptying is unclear. Nevertheless, because of the action on slowing gastric emptying and early case reports of pulmonary aspiration, the American Society of Anesthesiologists (ASA) recommended these drugs should be stopped either the day before the procedure (for those on once‐daily doses) or the week before (for those on weekly injections). This was, theoretically, to minimise the risk of incomplete gastric emptying leading to pulmonary aspiration on induction of anaesthesia [13]. However, several factors must be taken into consideration, including drug characteristics; the individual patient profile; the procedure; and the anaesthetic technique. Importantly, the adverse impact of drug cessation must also be considered (Table 2).

**Table 2:** Variables and risk factors, as well as potential outcomes, that need to be considered with respect to peri‐operative GLP‐1 receptor agonist management.

Variables and risk factors				Outcomes
Drug	Patient	Procedure	Anaesthesia	
Drug	Indication	Urgency	Technique	Pulmonary aspiration
Dose	Co‐morbidities	Nature	Airway	Glycaemic control
Route	Other drugs			Weight gain
Commencement	Fasting status			Complications of rapid sequence intubation
Cessation				

First, it is important to understand the data on gastric motility. There is clear evidence that GLP‐1 RAs are associated with delayed gastric emptying in most settings, as shown by: endoscopy [45, 46, 47, 48] (including capsular [49]); sonography [50, 51]; radiolabelled carbon ingestion [52, 53]; paracetamol ingestion [6, 7, 54, 55, 56, 57, 58, 59]; and scintigraphy [29, 60, 61]. However, most studies examine the impact of recent commencement of GLP‐1 RAs in volunteers, rather than long‐term use in the peri‐operative setting. Recent prospective data from the peri‐operative setting reflects this, highlighting an increased risk of residual gastric content with recent and ongoing use [62]. Data also suggest that there is tachyphylaxis with prolonged use of GLP‐1 RAs [29, 60] and, therefore, delayed gastric emptying might be of particular clinical relevance with recent commencement or dose escalation, although recent studies might call this into question in the peri‐operative setting [63]. The impact of longer‐term use of GLP‐1 RAs on people with diabetes mellitus or obesity needing anaesthesia for procedures remains to be clarified.

Most studies examining these concerns have reported surrogate outcomes. However, case reports describing clinically relevant evidence for delayed gastric emptying in appropriately starved patients either undergoing sedation or general anaesthesia for elective procedures [9, 11, 12, 64, 65, 66] or endoscopy [10, 67] (see online Supporting Information Table S1) have been published. Five of these report the duration of treatment, with four describing the recent commencement of GLP‐1 RAs, and all patients received this medication within the previous week. All patients had obesity, but all had appropriately fasted, and there were four cases of clinically demonstrated pulmonary aspiration. There were no other clear themes, and the sample size was too small to draw any meaningful conclusions. More case reports continue to be published, and although data from endoscopy settings might suggest that, in people with diabetes mellitus, GLP‐1 RAs might not necessarily increase the risk of pulmonary aspiration, there are no prospective, high‐quality studies in the peri‐operative setting to support this [68].

The pharmacokinetic and dynamic profile of GLP‐1 RAs must also be considered [15]. The half‐life of these drugs varies (Table 1), ranging from a few hours to a week. Thus, if a person who takes a 1 mg weekly dose of semaglutide for treatment of diabetes mellitus stops for one week, their plasma concentration will be the same as someone who is on 0.5 mg weekly and has not stopped. There are good data showing that the longer GLP‐1 RAs are ceased, the lower the likelihood of increased gastric residual gastric content [51]. However, even one week of cessation is associated with a high risk of residual gastric content in patients who fasted appropriately for surgery [51]. This evidence, as well as the case reports of patients withholding GLP‐1 RAs for a full week [64], suggests that this is also clearly insufficient duration of cessation to reduce the risk of pulmonary aspiration. Recent data even suggest that withholding GLP‐1 RAs for up to three weeks is also associated with increased gastric volume [69].

The individual patient profile is clearly relevant. All people taking GLP‐1 RAs have at least two risk factors for delayed gastric emptying: the drug plus either diabetes mellitus or obesity (or both). Indeed, six of the ten published case reports included people with at least three different risk factors for pulmonary aspiration (see online Supporting Information Table S1), which includes other conditions or drugs that may delay gastric emptying. Similarly, the nature of the procedure must also be considered, as emergency surgery may itself be a risk factor for pulmonary aspiration. Procedures in which sedation or no tracheal intubation are used, may also pose greater risks of pulmonary aspiration.

There are also other considerations with respect to a potentially full stomach. Firstly, the presence of gastric content, whilst a pre‐requisite, does not in itself lead to pulmonary aspiration but is one of many variables. Indeed, some data suggest that 6–16% of patients who appropriately followed fasting guidelines for elective surgery have gastric content or volumes associated with a higher risk of aspiration [70, 71, 72]. Importantly, these data also underscore the utility of point‐of‐care gastric ultrasound to facilitate risk stratification and appropriate intervention to avoid adverse outcomes. Whilst not yet used widely, there will increasingly be a role for point‐of‐care ultrasound in peri‐operative management.

The decision to perform rapid sequence intubation for patients with a presumed full stomach also carries additional potential risks, including difficult and failed tracheal intubation; oesophageal intubation; anaphylaxis; awareness under general anaesthesia; and airway trauma. Moreover, there are other interventions that can be implemented to reduce the risk of pulmonary aspiration, and rapid sequence intubation is merely one intervention. Others include: administration of prokinetics (e.g. erythromycin 3 mg.kg^‐1^ 1–2 h before induction) [73]; selecting a tracheal tube rather than a supraglottic airway device; correct use of cricoid force; head‐up positioning; and the use of orogastric or nasogastric tubes before induction of anaesthesia and tracheal extubation. There are no data available to objectively provide evidence for safety or efficacy of prolonged fasting times. In general, there are features that may be associated with an increased risk of full stomach and therefore, potential pulmonary aspiration (see online Supporting Information Table S2).

On the other hand, the impact of stopping these drugs must also be considered. Peri‐operative hyperglycaemia is associated with harm [74], including (but not limited to): increased length of hospital stay; surgical site infection; acute kidney injury; acute coronary syndrome; critical care admission; or time on a ventilator [75, 76]. In addition, if an individual is scheduled for the day of surgery admission, a raised blood glucose concentration may risk delay or cancellation of surgery, with the subsequent loss of surgical capacity. All these complications might result in increased cost and reduced bed capacity and impact on the healthcare economy.

The UK guideline for the peri‐operative management of people with diabetes mellitus undergoing surgery contains practical guidance on the pre‐operative manipulation of all diabetes mellitus medications, including on the day before or the day of surgery, whether in the morning or the afternoon [19]. In people without diabetes mellitus and who are using these drugs for weight loss, there is potentially an increased risk of developing peri‐operative hyperglycaemia [77], though further data are required.

Stress hyperglycaemia is a transiently raised blood glucose concentration in people not known previously to have diabetes mellitus. It occurs most frequently in people at risk of developing type 2 diabetes mellitus, including, but not limited to: people living with obesity; a previous history of gestational diabetes mellitus; a family history of diabetes mellitus; long‐term glucocorticoid use; age > 40 y; or people of South Asian ancestry. People who develop stress hyperglycaemia have more adverse peri‐operative outcomes than those with diabetes mellitus [78, 79, 80]. People not known to have diabetes mellitus but who develop hyperglycaemia do not have their glucose tested as frequently as those with diabetes mellitus, nor is intervention started despite poor glycaemic control [16, 81]. Thus, people using a GLP‐1RA for obesity are at risk of stress hyperglycaemia and should have regular capillary glucose measurements with prompt intervention, should hyperglycaemia occur. The impact that GLP‐1RA use, or of cessation and recommencement of GLP‐1 RAs in people with obesity on the risk of developing stress hyperglycaemia, remains unclear.

Given the complexities highlighted, recommendations on pre‐operative timing of cessation alone are probably not sufficient to manage peri‐operative risk in patients receiving anaesthesia for procedures (Box 1). However, the interplay between variables, risk factors and patient outcomes is complex.

Box 1 **Recommendations for the peri‐operative management of patients taking glucagon‐like peptide‐1 receptor agonists and glucose‐dependent insulinotropic peptide.**

- The risk of pulmonary aspiration and mitigation strategies should be discussed with the patient using a shared decision‐making approach.
- Patients should continue to take glucagon‐like peptide‐1 receptor agonists throughout the peri‐operative period.
- Patients and clinicians should adhere to recommended fasting guidelines.
- Upper gastrointestinal symptoms alone should not be used to determine gastric content.
- Regional anaesthesia should be considered as the primary anaesthetic technique, if appropriate.
- Point‐of‐care gastric ultrasound should be considered before induction of anaesthesia to facilitate risk stratification, if appropriate.
- Individualised pulmonary aspiration risk assessment should be completed, accounting for drug, patient and procedural factors.
- Anaesthesia and airway management should aim to reduce the risk of pulmonary aspiration on induction of anaesthesia, during maintenance and after emergence of anaesthesia. This could include: administering prokinetics; using a tracheal tube; modified rapid sequence intubation (with or without cricoid force, depending on local practice); head‐up position for induction of anaesthesia; potential use of gastric tubes to empty the stomach before induction of anaesthesia and tracheal extubation; and awake tracheal extubation.

## Sodium‐glucose cotransporter‐2 inhibitors

Sodium‐glucose cotransporter‐2 inhibitors lower blood glucose by inhibiting reabsorption from the proximal convoluted tubules and inducing glycosuria, mimicking starvation [82] (Table S3). In healthy individuals, glucose is the main driver for insulin secretion, and if glucose concentrations drop due to glycosuria, the drive to secrete insulin falls. If there is no insulin, the ability for glucose to enter cells also reduces. Cells, therefore, require an alternative energy substrate, i.e. ketones. As insulin concentrations reduce, glucagon concentrations increase. This change in the insulin/glucagon ratio allows free fatty acids to be liberated from adipose tissue, which are then taken to the liver and converted to ketones [82]. Hence even in healthy individuals, starvation leads to ketosis, with concentrations rising above 6 mmol.l^‐1^ if carbohydrate restriction is prolonged [83]. However, in people who are otherwise well and without concomitant diabetes mellitus, these concentrations are not dangerous because the time taken to reach these concentrations allows for adequate renal and respiratory compensation to prevent metabolic acidosis.

Sodium‐glucose cotransporter‐2 inhibitors have been used in the management of diabetes mellitus for several years, but with the additional cardiovascular benefits they are now co‐first line with metformin in the treatment algorithm for people at high cardiovascular risk or with existing atherosclerotic disease, heart failure or chronic kidney disease [84, 85, 86].

Sodium‐glucose cotransporter‐2 inhibitors‐induced glycosuria is accompanied by an osmotic diuresis [87]. This leads to a decrease in circulating volume, a lowering of blood pressure and a reduction in cardiac workload and glomerular pressure. Emerging data also suggest that low‐grade hyperketonaemia leads to improved cardiac myocyte mitochondrial efficiency and function [88, 89]. Taken together, they have positive effects on heart failure and help to prevent the progression of chronic kidney disease and are licensed for these indications in people with or without diabetes mellitus [3].

These drugs all have a half‐life of 12–13 h and do not have any active metabolites. However, unusually, their pharmacodynamic effect on urinary glucose excretion lasts significantly beyond effective plasma drug concentrations in people with or without type 2 diabetes mellitus. Although, 24 h after cessation there is only 25% of the plasma drug concentration remaining, there does continue to be increased urinary glucose excretion beyond 48 h. If sodium‐glucose cotransporter‐2 inhibitors are stopped for 72 h, < 1% remains in the circulation and urinary glucose excretion normalises to baseline. Theoretically, the pharmacodynamic effect on urinary glucose excretion would be associated with the changes in insulin/glucagon ratio, and therefore risk of ketone generation may be better linked to the pharmacodynamic effects rather than plasma drug concentrations. In the peri‐operative setting, there are differing recommendations on when these drugs should be stopped pre‐operatively. Medicines and Healthcare Products Regulatory Agency (MHRA) guidance suggests these drugs should be omitted the day before planned major surgery, whilst the guidance from the US Food and Drug Administration recommends considering cessation 3–4 days before any scheduled surgery [19, 85].

The major concern about the peri‐operative use of SGLT2 inhibitors is the development of diabetic ketoacidosis (DKA) and particularly euglycaemic DKA and ketoacidosis in those with or without diabetes mellitus, where the glucose concentrations are normal (i.e. < 11.0 mmol.l^‐1^). With the lowering of glucose concentrations, and the resultant change in the insulin/glucagon ratio and the predisposition to develop ketosis, any increase in physiological stress that leads to the production of counterregulatory hormones further drives the development of hyperketonaemia. Because of the speed of increased ketone concentrations and the inability to compensate for the resulting acidaemia, ketone concentrations can rise quickly to > 3.0 mmol.l^‐1^, and the pH may drop to < 7.3 which is the threshold for defining DKA [90]. The ‘D’ of DKA means that glucose may be > 11.0 mmol.l^‐1^ or there is a history of diabetes mellitus [91].

Data suggest that the risk of peri‐operative DKA in those patients taking an SGLT2 inhibitor is higher than in those who are not (1.02 vs. 0.69 per 1000 patients, OR 1.48, 95%CI 1.02–2.15, p = 0.037) [92]. A further review of almost 100 cases of peri‐operative DKA suggested that omitting the drug > 2 days pre‐operatively did not result in DKA occurring [93]. Other work has shown that peri‐operative SGLT2 inhibitor cessation is associated with a reduced risk of high anion gap acidosis [94]. Data also suggest that the incidence of peri‐operative ketoacidosis is greater in those patients having emergency surgery than elective surgery (1.1% vs. 0.17%) [95], whilst in the non‐operative setting, is greater in patients with diabetes mellitus than those without (1 in 339 vs. 1 in 15,592) [96, 97]. However, these data may be associated with significant confounding and routine application of results to change clinical practice should be avoided.

Until recently, it was thought that people without diabetes mellitus had sufficient insulin concentrations to prevent significant ketosis, but two recent publications have challenged this [98, 99]. None of the four patients described in these reports had diabetes mellitus, and thus they had euglycaemic ketoacidosis. The pathophysiology remains the same, and as a result, clinicians need to remain vigilant for this rare condition in those patients without diabetes mellitus but who continue to take SGLT2 inhibitors peri‐operatively.

The same argument made for cessation of GLP‐1RAs and the resultant harms from peri‐operative hyperglycaemia can also be made with cessation of SGLT2 inhibitors. Moreover, data suggest that cessation of SGLT2 inhibitors in people receiving them for heart failure may be associated with worsening of heart failure [100]. Thus, there needs to be a balance between stopping the drugs early to prevent the risk of ketoacidosis, against the risks of deferring or cancelling a procedure due to pre‐operative hyperglycaemia and risk of postoperative complications. Given the evidence and pharmacokinetic/pharmacodynamic profiles, as well as a pragmatic approach, previous UK guidelines of omission the day before surgery remain acceptable. If the drug is taken in the morning, and the procedure is in the morning, that would equate to at least a 48‐hour gap – with it being over 52 hours if it was an afternoon procedure (Box 2). If the individual takes the drug in the evening, it is still a minimum of a 36‐hour gap.

Box 2 **Recommendations for the peri‐operative management of patients taking sodium‐glucose cotransporter‐2 inhibitors.**

- The risk of peri‐operative ketoacidosis and mitigation strategies should be discussed with the patient using a shared decision‐making approach.
- Sodium‐glucose cotransporter‐2 inhibitors should be omitted the day before and the day of a procedure.
- Patients and clinicians should adhere to recommended fasting guidelines and avoid prolonged starvation times.
- For patients discharged from hospital on the day of surgery, sodium‐glucose cotransporter‐2 inhibitors should be restarted once eating and drinking normally (usually 24–48 h after surgery).
- For patients staying in hospital after surgery, consider restarting sodium‐glucose cotransporter‐2 inhibitors once eating and drinking normally and capillary ketones are < 0.6 mmol.l^‐1^.
- For patients diagnosed with diabetes mellitus on a very low energy/liver reduction diet for the purposes of surgery, sodium‐glucose cotransporter‐2 inhibitors should be stopped at commencement of the diet, and adjust diabetes mellitus treatment as necessary.
- Written sick‐day rules should be provided to patients at pre‐operative assessment and at discharge.

There are reports of postoperative ketoacidosis occurring even when patients have withheld SGLT2 inhibitors for > 72 h, emphasising that the risk of complications is a continuum rather than having a defined threshold when ketoacidosis will not occur. Importantly, mitigations to complications during the time of withholding SGLT2 inhibitors may be beneficial, such as ensuring patients remain well hydrated, avoid long starvation periods, and adequate glucose and ketone monitoring. In settings of unplanned or unavoidable prolonged fasting, there may be safety benefits to considering glucose‐containing intravenous fluids to mitigate ketone generation.

Before certain types of surgery, including bariatric and some laparoscopic procedures, patients may be started on a very low‐energy diet, also known as a liver reduction diet [101]. This diet is mainly milk, yoghurt and soup‐based and generally consists of 800–1000 calories per day, whilst being very low in carbohydrate and fat content. The aim is to decrease the size of the liver by forcing the body to utilise the stored carbohydrates within the liver. This ensures maximal surgical working space, results in a softer, more mobile liver and is thought to increase patient safety. Typically, patients are on the pre‐operative very low‐calorie diet for two weeks, but some centres advocate up to four weeks in people with very high body mass index [102]. As well as potentially reducing insulin resistance and improving glycaemic control [103], this diet results in a degree of ketosis on its own, and as a result, the continuation of SGLT2 inhibitors in people with diabetes mellitus could lead to significant ketoacidosis [103, 104]. Thus, this cohort of patients requires specific consideration.

Sick‐day rules (also known as sick‐day medication guidance) are adjustments that need to made to medicines to reduce the risk of adverse medicine‐related events during acute illness, and involve either withholding or dose adjustment of treatment [105]. People receiving SGLT2 inhibitors for treatment of diabetes mellitus may be familiar with them, but in the peri‐operative setting, it is important that specific sick‐day rules are provided to ensure safe recovery. This information could include general advice for managing diabetes mellitus; complication risk‐reduction strategies; information on symptoms and signs of DKA; what to do in the event of postoperative illness; specifics of SGLT2 inhibitors dose management, and contact information for assistance.

## Discussion

Given the dearth of high‐quality peri‐operative studies, there remains uncertainty regarding optimal peri‐operative management of patients taking GIP/GLP‐1 RAs and SGLT2 inhibitors. This consensus statement synthesises the existing evidence and provides pragmatic recommendations to support clinicians and patients with shared decision‐making (see online Supporting Information Appendix S1). In patients taking GIP/GLP1‐RAs, continuation of treatment is currently recommended, but should be supported through risk assessment and stratification, shared decision‐making and the use of peri‐operative techniques to mitigate risk of pulmonary aspiration. Patients taking SGLT2 inhibitors should stop these medications one day pre‐operatively. Whilst we focus on the peri‐operative use of individual drugs, there are a multitude of factors that may adversely affect risk of pulmonary aspiration, glycaemic dysregulation, cardiac, renal and glycaemic deterioration and ketotic states; clinicians should take each of these into account in delivering individualised care. Finally, we focus on elective peri‐operative care, but in the setting of emergency surgery, endoscopy, and other settings, recommendations may be less applicable.

The limitations of these recommendations are due largely to the limited evidence base. Studies to date are predominantly case reports or retrospective clinical trials with potential for bias. We recognise that the evidence in this field continues to evolve and therefore these recommendations are only valid at the time of writing. As the adverse outcomes of interest occur with relative infrequency, definitive conclusions will likely only be reached through large prospective observational studies. We have an opportunity to develop such datasets through collaboration and effective use of data sharing. Furthermore, the use of these drugs also varies with time, and some people might not report using GIP/GLP‐1 RAs as they are now able to acquire them without physician prescription [106, 107]. Therefore, the frequency at which clinicians are likely to meet people taking these medications will probably increase in the future. Moreover, certain specific elements of management are difficult to make explicit recommendations based on no evidence, as well as the need to be sensitive to institutional capability and capacity. For example, we are unable to recommend a frequency of ketone monitoring for people on SGLT2 inhibitors, and local providers will need to consider this according to their own setting.

Future research is required to explore the risk of pulmonary aspiration and guide GIP/GLP‐1 RA commencement and cessation, pre‐operative fasting times, and risk‐mitigating strategies. Research is also required to describe the optimal timing of cessation of SGLT2 and the associated risk of ketoacidosis in people with and without diabetes mellitus.

This pragmatic, multidisciplinary consensus statement aims to support decision‐making and management of patients taking GIP/GLP‐1 RAs or SGLT2 inhibitors during peri‐operative care. Although further evidence is awaited, it is hoped that these recommendations facilitate shared decision‐making between clinicians and patients, improve safety and result in a standardised approach in the UK which can then be evaluated through large national observational studies.

## Supporting information


**Appendix S1.** Patient summary of guidance.

**Table S1.** Case reports of delayed gastric emptying in peri‐operative settings.
**Table S2.** Risks of pulmonary aspiration.
**Table S3.** Generic and trade names of GLP‐1 receptor agonists and SGLT2 inhibitors.
