# Supplementary material for: Elective peri‐operative management of adults taking glucagon‐like peptide‐1 receptor agonists, glucose‐dependent insulinotropic peptide agonists and sodium‐glucose cotransporter‐2 inhibitors: a multidisciplinary consensus statement: A consensus statement from the Association of Anaesthetists, Association of British Clinical Diabetologists, British Obesity and Metabolic Surgery Society, Centre for Perioperative Care, Joint British Diabetes Societies for Inpatient Care, Royal College of Anaesthetists, Society for Obesity and Bariatric Anaesthesia and UK Clinical Pharmacy Association
Source: Anaesthesia. 2025 Jan 9;80(4):412–24. doi: 10.1111/anae.16541 (PMC11885194; doi:10.1111/anae.16541)
Supplement: Supplementary file 1 — Appendix S1. Patient summary of guidance. [file ANAE-80-412-s001.pdf]

This is a summary of the recommendations on the management of glucagon-like peptide-1 receptor agonists and sodium-glucose cotransporter-2 inhibitors and preparing for surgery. Be sure to talk to your healthcare team so they can help you make a clear, personal plan for how to handle your medicines before, during, and after surgery.

The full recommendations can be found by clicking here.

Further research and evidence in the future is likely to improve our understanding on the use of these medicines. Therefore, advice is likely to change as more knowledge becomes available.

Information about taking glucagon-like peptide-1 (GLP-1) receptor agonists and preparing for surgery

Background

- GLP-1 and GIP are hormones released from the lining of the gut when sugars are detected as food is digested
- These hormones also slow down stomach emptying
- Medicines like Ozempic, Wegovy or Mounjaro act like GLP-1 hormones
- They can be prescribed for the treatment of diabetes and for weight loss

GLP-1 agonists and surgery

- Around the time of surgery these medicines can have some rare risks.
- We need to balance the risk of stopping or continuing these medicines before planned surgery.

Possible harms of not stopping these medicines before surgery

- When people have a general anaesthetic or sedation the muscles in the body become relaxed and reflexes that usually protect our lungs, like coughing, are not as effective.
- If there is a full stomach, contents can be vomited and inhaled, which can cause infection or inflammation of the lungs and problems with breathing.
- This is called **pulmonary aspiration**.
- It is a rare but serious complication.

Your anaesthetist can do certain procedures to reduce the risk of pulmonary aspiration. This might include:

- Pressure on the front of the neck when drifting off to sleep
- Medicines that speed up stomach emptying
- Using a breathing tube that protects the lungs
- Not lying completely flat when having an anaesthetic
- A tube through the nose or mouth to drain the stomach before an anaesthetic or before being woken up
- Ultrasound scan of the stomach before an anaesthetic
-

Possible harms of stopping these medicines before surgery

Stopping these medicines can lead to high blood sugar levels. High blood sugar levels can mean:

- Slower recovery
- Infections
- Heart complications
- Needing admission to intensive care
- Postponing surgery

What to do before surgery

If you take a GLP-1 receptor agonist like Ozempic, Wegovy or Mounjaro, and are due to have surgery, healthcare professionals will make a plan with you. The plan will depend on the type of medication, why you take it and other health conditions and what procedure and anaesthetic you will be having.

The risk should be weighed up against the potential harm of stopping the medication.

- Discuss your risk and options with an anaesthetist

- You should continue to take GLP-1 RAs before and after surgery
- You should follow fasting instructions given before surgery
- Where it is possible, your anaesthetist may suggest you have the procedure awake. For example, with regional block or spinal anaesthetic

Information about taking sodium-glucose cotransporter-2 inhibitors (SGLT2is) and preparing for surgery

Background

- SGLT2is lower blood sugar by helping the kidneys clear excess sugar
- Medicines like empagliflozin and dapagliflozin are SGLT2is
- They can be prescribed for the treatment of diabetes, heart failure and kidney disease

SGLT2is and surgery

- Around the time of surgery these medicines can have some rare risks.
- We need to balance risk of stopping or continuing these medicines before planned surgery

Possible harms of not stopping these medicines before surgery

- When fasting for surgery, sugar cannot be used for energy, so the body breaks down fat instead
- This process produces toxic acids called ketones
- In people taking SGL2i these toxic acids can build up and cause harm
- This is called **ketoacidosis**.
- This is a rare but serious complication.

Possible harms of stopping these medicines before surgery

Stopping these medicines can lead to high blood sugar levels. High blood sugar levels can mean:

- Slower recovery
- Infections

- Heart complications
- Needing admission to intensive care unit
- Postponing surgery

What to do before surgery

- Do not take the SGLT-2i the day before and the day of surgery
- Follow fasting instructions before surgery
- Drink plenty of fluids the day before surgery
- Restart these medicines at home after surgery once you are eating and drinking normally. You may need some blood tests before restarting them if you stay in hospital after surgery.
- Follow the sick day rules you have received for these medicines.
- If you are on a special diet, such as a very low energy diet, you may be advised to stop your medicines earlier.

GLUCAGON-LIKE PEPTIDE-1 RECEPTOR AGONISTS (GLP-1 RA) AND PREPARING FOR SURGERY

▷ Medicines like Ozempic, Wegovy or Mounjaro can slow down the stomach emptying
▷ Taking them around the time of surgery can have some rare risks
▷ We need to balance risk of stopping or continuing these medicines before planned surgery

POSSIBLE HARMS OF NOT STOPPING THESE MEDICINES

▷ General anaesthetic or sedation make muscles relax
▷ Reflexes that protect our lungs, like coughing, are not as strong
▷ With a full stomach, contents can be vomited and inhaled
▷ This can cause problems with breathing
▷ This is called pulmonary aspiration
▷ It is a **rare** but serious complication

POSSIBLE HARMS OF STOPPING THESE MEDICINES

▷ Stopping these medicines can lead to high blood sugar
▷ High blood sugar around the time of surgery can mean:
- ▷ Slower recovery
- ▷ Infections
- ▷ Heart complications
- ▷ Needing admission to intensive care
- ▷ Postponing surgery

WHAT TO DO BEFORE SURGERY

1. CONTINUE TAKING YOUR GLP-1 RA MEDICINE AS USUAL
2. FOLLOW FASTING INSTRUCTIONS
3. DISCUSS WITH THE ANAESTHETIST: RISKS AND OPTIONS

SODIUM-GLUCOSE COTRANSPORTER-2 (SGLT-2) INHIBITORS AND PREPARING FOR SURGERY

- ▷ Medicines like empagliflozin and dapagliflozin are used to improve blood sugar and blood pressure
- ▷ Taking them around the time of surgery can have some rare risks
- ▷ **We need to balance risk of stopping or continuing these medicines before planned surgery**

POSSIBLE HARMS OF NOT STOPPING THESE MEDICINES

- ▷ If you continue taking these medicines before surgery, there is a risk of ketoacidosis
- ▷ This is a **rare** but serious complication

POSSIBLE HARMS OF STOPPING THESE MEDICINES

- ▷ Stopping these medicines can lead to high blood sugar
- ▷ High blood sugar around the time of surgery can mean:
  - ▷ Slower recovery
  - ▷ Infections
  - ▷ Heart complications
  - ▷ Needing admission to intensive care
  - ▷ Postponing surgery

WHAT TO DO AROUND SURGERY

1. DO NOT TAKE SGLT2 INHIBITOR THE DAY BEFORE AND THE DAY OF SURGERY
2. FOLLOW FASTING INSTRUCTIONS
3. DRINK PLENTY OF FLUIDS THE DAY BEFORE SURGERY
4. RESTART THESE MEDICINES AT HOME AFTER SURGERY ONCE YOU ARE EATING AND DRINKING NORMALLY
5. FOLLOW THE SICK DAY RULES YOU HAVE RECEIVED FOR THESE MEDICINES
