# Supplementary material for: Elective peri‐operative management of adults taking glucagon‐like peptide‐1 receptor agonists, glucose‐dependent insulinotropic peptide agonists and sodium‐glucose cotransporter‐2 inhibitors: a multidisciplinary consensus statement: A consensus statement from the Association of Anaesthetists, Association of British Clinical Diabetologists, British Obesity and Metabolic Surgery Society, Centre for Perioperative Care, Joint British Diabetes Societies for Inpatient Care, Royal College of Anaesthetists, Society for Obesity and Bariatric Anaesthesia and UK Clinical Pharmacy Association
Source: Anaesthesia. 2025 Jan 9;80(4):412–24. doi: 10.1111/anae.16541 (PMC11885194; doi:10.1111/anae.16541)
Supplement: Supplementary file 2 — Table S1. Case reports of delayed gastric emptying in peri‐operative settings. Table S2. Risks of pulmonary aspiration. Table S3. Generic and trade names of GLP‐1 receptor agonists and SGLT2 inhibitors. [file ANAE-80-412-s002.docx]

**Table S1.** Case reports of delayed gastric emptying in peri-operative settings.

| **Year** | **Author** | **Setting** | **Indication for GLP-1 RA** | **Risk factors** | **GLP-1 RA** | **Duration of treatment** | **Cessation** | **Fasting** | **Outcome** | **Evidence of pulmonary aspiration** | **Timing of aspiration** | **Airway during aspiration** |
| --- | --- | --- | --- | --- | --- | --- | --- | --- | --- | --- | --- | --- |
| 2024 | Avraham | Elective endoscopic retrograde cholangiopancreatography; general anaesthesia | Diabetes, obesity | Diabetes, obesity (BMI 35 kg/m2), choledocholithiasis | Semaglutide 1 mg weekly | Not defined | 6 days pre-procedure | > 12 hours | Regurgitation on induction of anaesthesia | Yes | Induction | None |
| 2024 | Avraham | Elective breast abscess; general anaesthesia | Obesity | Obesity (BMI 32 kg/m2) | Semaglutide 1 mg weekly | Not defined | 4 days pre-procedure | > 8 hours | After SAD removal, regurgitation of solid and liquid content | Yes | Extubation | None |
| 2023 | Beam | MRI; sedation | Obesity | Obesity (BMI 28 kg/m2) | Semaglutide weekly (dose not defined) | 1 month | 7 days pre-procedure | 18 hours | Gastric ultrasound demonstrating full stomach; cancelled procedure | No | No | No |
| 2023 | Beam | Robot-assisted hysterectomy; general anaesthesia | Diabetes, obesity | Diabetes, obesity (BMI 37.7 kg/m2), GORD, chronic pain (on opioids) | Tirzepatide 12.5 mg (dosing regimen not defined) | Not defined | 2 days pre-procedure | Previous night | Vomiting large volumes before tracheal extubation; no aspiration as still intubated | No | No | No |
| 2023 | Fujino | Endoscopy; sedation | Obesity | Obesity (BMI 45.6 kg/m2), diabetes | Semaglutide 0.25 mg weekly | 1 month | Not ceased | 10 hours | Large amount of food in gastric body | No | No | No |
| 2024 | Girón-Arango | Urologic procedure; general anaesthesia | Diabetes, obesity | Diabetes, obesity (BMI 36 kg/m2) | Semaglutide 1 mg weekly | Not defined | 1 day pre-procedure | Solids, 14 hours, fluids, 5 hours | Gastric ultrasound demonstrating full stomach; proceed with procedure under spinal | No | No | No |
| 2023 | Gulak | Lumpectomy, axillary node dissection, reduction mammoplasty; general anaesthesia | Obesity | Obesity (BMI 28 kg/m2) | Semaglutide 0.5 mg weekly | 5 months | 2 days pre-procedure | Solids, 20 hours; fluids, 8 hours | Regurgitation on induction of anaesthesia; no evidence of aspiration | No | No | No |
| 2023 | Hodgson | Shoulder arthroscopy; general anaesthesia | Diabetes, obesity | Diabetes, obesity (BMI 42.9 kg/m2), GORD | Semaglutide 2 mg weekly | Not defined | 3 days pre-procedure | > 10 hours | Substantive volume of gastric contents was suctioned after securing the airway, despite RSI done | No | No | No |
| 2023 | Kittner | Total knee arthroplasty; general anaesthesia | Diabetes, obesity | Diabetes, obesity | Semaglutide (dose not defined) | Not defined | 1 day pre-procedure | Solids, 11 hours | Pre-procedural gastric content demonstrating solid food products; case postponed | No | No | No |
| 2023 | Kittner | Knee arthroscopy; general anaesthesia | Diabetes, obesity | Diabetes, obesity (class 1) | Semaglutide (dose not defined) | Not defined | 6 days pre-procedure | Solids, 10 hours | Pre-procedural gastric content demonstrating solid food products; case postponed | No | No | No |
| 2023 | Kittner | Total knee arthroplasty; general anaesthesia | Diabetes, obesity | Diabetes, obesity (class 2) | Semaglutide (dose not defined) | Not defined | 1 day pre-procedure | Solids, 14 hours | Pre-procedural gastric content demonstrating solid food products; case postponed | No | No | No |
| 2023 | Klein | Endoscopic ablation of Barrett's oesophagus; sedation | Obesity | Obesity (BMI 37 kg/m2), GORD, Barrett's oesophagus, previous aspiration lung abscess | Semaglutide 1.7 mg weekly | 2 months | Not defined | > 18 hours | Large quantities of liquid and solid material in stomach on endoscopy, intubated | Yes | Maintenance | None |
| 2024 | Milne | Elective total knee surgery; general anaesthesia | Not defined | Not defined | Semaglutide (dose not defined) | Not defined | Not defined | Not defined | Aspirates in orpharynx during bag-mask ventilation | Yes | Induction | Bag-mask |
| 2024 | Milne | Elective total knee surgery; general anaesthesia | Not defined | Not defined | Semaglutide (dose not defined) | Not defined | Not defined | Not defined | After SAD placement; conversion to tracheal intubation because of tracheal soiling | Yes | Maintenance | SAD |
| 2024 | Milne | Elective total knee surgery; general anaesthesia | Not defined | Not defined | Semaglutide (dose not defined) | Not defined | Not defined | 13 hours | Large particles of undigested food regurgitated into the pharynx after extubation | Yes | Extubation | None |
| 2024 | Queiroz | Renal nodule ablation; general anaesthesia | Obesity | Obesity (BMI 31.2 kg/m2) | Semaglutide 0.5 mg weekly | 6 days | 6 days pre-procedure | Solids, 9 hours; fluids 9 hours | Incidental finding of 931.8 ml of gastric content on CT; gastric tube inserted but thick fluid therefore drainage unsuccessful | No | No | No |
| 2023 | Weber | Hysteroscopy; general anaesthesia | Obesity | Obesity (BMI not defined) | Tirzapetide (dose not defined) | Recent (exact time not defined) | Not defined | Not defined | Aspirates in oropharynx after SAD inserted, replaced by tracheal tube; large emesis after intubation of undigested food and thick aspirate (500 ml) | Yes | Maintenance | SAD |
| 2023 | Wilson | Foot arthrodesis; block; sedation (propofol and ketamine) | Diabetes, obesity | Obesity (BMI 48.4 kg/m2), diabetes | Dulaglutide 1.5 mg weekly | Not defined | Within the past week | Solids, 10 hours; fluids 4 hours | Secretions in oropharynx, converted to general anaesthesia, after tracheal intubation, bilious particulate matter suctioned | No | No | No |
| 2023 | Wilson | Thyroidectomy; general anaesthesia | Diabetes, obesity | Obesity (BMI 50.1 kg/m2), diabetes, GORD | Semaglutide 7 mg daily (oral) | Not defined | 2 days pre-procedure | Solids, 16 hours; fluids 5 hours | After extubation, projectile vomiting of 300 ml bile-tinted particulate matter; no evidence of aspiration | No | No | No |

**Table S2.** Factors that might increase the risk of pulmonary aspiration that might be considered in risk stratifying individual patients, along with examples.

|  | **Risk factor** | **Example** |
| --- | --- | --- |
| *Drug* | Recent commencement | Started within 3 months |
|  | Dosing within 5 half-lives | Dose received within a week |
| *Patient* | BMI >30 kg.m^-2^ | Morbid obesity |
|  | Evidence of gastroparesis | Diabetes with evidence of neuropathy |
|  | Other risk factors for delayed gastric emptying | Opioid use, renal failure |
|  | Short duration of fasting | Fasting for <8 hours |
| *Procedure* | Urgent surgery | Procedures requiring surgery within 48 hours |
|  | Risk of aspiration | Laparoscopic surgery |

**Table S3.** Generic and trade names of commonly used glucagon-like peptide-1 receptor agonists (GLP-1 RA), glucose-dependent insulinotropic peptide (GIP) agonists and sodium-glucose cotransporter-2 inhibitors (SGLT-2is).

| **Class** | **Drug name** | **Trade name** |
| --- | --- | --- |
| GLP-1 RA | Exanetide | Byetta |
|  | Lixisenatide | Lyxumia |
|  | Liraglutide | Victoza |
|  | Abiglutide | Eperzan |
|  | Dulaglutide | Trulicity |
|  | Semaglutide | Ozempic, Wegovy |
| GLP-1 RA/GIP | Tirzapatide | Mounjaro |
| SGLT-2i | Dapagliflozin | Forxiga |
|  | Canagliflozin | Ivokana |
|  | Empagliflozin | Jardiance |
|  | Erugliflozin | Steglatro |
